# The effectiveness of vibrational stimulus to accelerate orthodontic tooth movement: a systematic review

**DOI:** 10.1186/s12903-017-0437-7

**Published:** 2017-12-01

**Authors:** Dian Jing, Jiani Xiao, Xiaobing Li, Yu Li, Zhihe Zhao

**Affiliations:** 1 State Key Laboratory of Oral Diseases & National Clinical Research Center for Oral Diseases, Department of Orthodontics, West China Hospital of Stomatology, Sichuan University, Chengdu, China; 2 State Key Laboratory of Oral Diseases & National Clinical Research Center for Oral Diseases, Department of Pediatric Dentistry, West China Hospital of Stomatology, Sichuan University, Chengdu, China

**Keywords:** Accelerate, Orthodontics, Tooth movement, Vibration

## Abstract

**Background:**

In recent years, it has been a hot research topic to accelerate orthodontic tooth movement (OTM) through vibration. This review was therefore aimed to systematically evaluate the available evidences on the efficacy of vibrational stimulus to accelerate OTM.

**Methods:**

Randomized controlled trials and controlled clinical trials that evaluated the efficacy of vibration on OTM acceleration were searched through electronic and manual search. Two review authors independently conducted the study inclusion, quality assessment and data extraction. The quality of synthesized evidence was assessed according to GRADE system.

**Results:**

Eight clinical trials were included in this systematic review. Four studies found that vibration did not enhance the rate of OTM during alignment phase. Two studies revealed that the use of vibratory stimulation accelerated canine retraction. No deleterious effects including pain perceptions and root resorptions were reported.

**Conclusions:**

Within the limitations of this review, weak evidence indicates that vibrational stimulus is effective for accelerating canine retraction but not for alignment. The effects of vibration on pain intensity and root resorption during orthodontic treatment are inconclusive. Future high-quality clinical trials are needed before warranting recommendations to clinical application.

## Background

The length of orthodontic treatment, which normally ranges from 24 to 36 months, is one of the main concerns to patients [1, 2]. The prolonged treatment duration could reduce the compliance of patients, and cause numerous adverse effects including white spot lesions, periodontal diseases and external root resorptions [3, 4]. Therefore, approaches to speed up orthodontic tooth movement (OTM) and the resulting reduction of treatment duration are always desirable to orthodontists and patients.

In recent years, numerous surgical and nonsurgical adjunctive procedures to accelerate OTM have been introduced [5–7]. Surgical techniques like corticotomy have been reported to facilitate tooth movement in short term via inducing regional acceleratory phenomenon [5, 7]. However, the invasiveness and postoperative discomfort make patients less receptive to these techniques and restrict the routine application in clinics [8]. Several nonsurgical adjuncts including laser therapy, electric current, pulsed electromagnetic fields and photobiomodulation are suggested to promote tooth movement [9–11]. However, the use of these approaches is also limited since the necessity of performance by disciplined clinicians and low quality of evidence [5, 9].

Among the nonsurgical interventions, vibrational stimulus is promising and has already been commercial since portability, convenience and invasiveness. The potency of applying vibrational stimulus to accelerate OTM has been identified in previous animal studies [12, 13]. Recently, several clinical reports have investigated the effects of vibrations on the rate of tooth movement [14–23]. Nevertheless, the methodological heterogeneity and inconclusive results of these studies could cause difficulties to evaluate evidences and mislead clinical practice. Therefore, a comprehensive systematic review addressing the effectiveness of vibration to accelerate OTM would be beneficial to practitioners.

In this study, we performed a critical systematic review on randomized controlled trials (RCT) and controlled clinical trials (CCT) to evaluate the efficacy of vibration in accelerating tooth movement in an evidence-based approach.

## Methods

This systematic review was carried out and reported according to Cochrane Handbook for Systematic reviews of Interventions [24] and Preferred Reporting Items for Systematic Reviews and Meta-analyses (PRISMA) [25]. Two review authors performed the literature search, study inclusion, data extraction and risk of bias assessment independently. Any discrepancy was resolved by reaching a consensus through discussion with a third reviewer.

### Eligibility criteria

The inclusion criteria were as follows: (1) The study should evaluate the effectiveness of vibrational stimulus on OTM; (2) Study design: the study should be RCT or CCT; (3) Participants: subjects should be systematically healthy patients who require orthodontic treatment; (4) Type of interventions: subjects should be assigned to experimental or control/placebo group based on receiving vibrational stimulus or not; (5) Type of outcomes: indicator of tooth movement velocity and related treatment parameters.

The exclusion criteria were as follows: (1) Retrospective design, cohort study, case reports, descriptive studies or letters; (2) Animal experiments; (3) Participants with systematic diseases that affect bone metabolism or orthodontic treatment.

### Information sources, search strategy, and study selection

PubMed, Embase, Cochrane Central Register of Controlled Trials (CENTRAL), and System for Information on Grey Literature in Europe (SIGLE) were searched for literature until Nov 2016. We adopted a combination of Medical Subject Headings (MeSH) with related free text words for the search in PubMed, and optimized the search strategy for each database respectively. The specific search strategies were presented in Table 1. In addition, a manual search was conducted among relevant journals and reference sections of retrieved records. The search was carried out in English without publication time limitation.

**Table 1:** Search strategies for Each Database

Step	PubMed	Embase, CENTRAL, & SIGLE
1	Vibration [Mesh] OR vibratory OR vibrational	vibration OR vibratory OR vibrational
2	Orthodontics [Mesh] OR orthodont*	Orthodontics OR orthodont*
3	Tooth movement [Mesh] OR move* OR retract*	tooth movement OR move* OR retract*
4	1 AND 2 AND 3	1 AND 2 AND 3

After the removal of duplicate records, the titles and abstracts of identified studies were screened to exclude irrelevant citations. The full-text of reserved studies were retrieved and assessed referring to eligibility criteria by two reviewers independently.

### Data items and collection

A customized form was developed for data extraction. The general information of recruited studies including author, publication year, country, demographic information, vibration parameters, type of tooth movement, follow-ups and results were extracted.

The primary outcome of interest in this review was the rate of tooth movement, time needed to complete a predefined tooth movement and accumulated moved distance. The secondary outcome included patients’ quality of life and adverse effects like root resorption and discomfort.

### Risk of bias in individual studies

The risk of bias of included trials were evaluated according to Cochrane Collaboration’s tool for assessing risk of bias [26]. This tool assesses the methodological quality of clinical trials through seven domains including random sequence generation, allocation concealment, blinding of participants and personnel, blinding of outcome assessment, incomplete outcome, selective reporting and other bias. The primary study was categorized as low risk when all items being assessed as low risk of bias, as unclear risk if one of more items being assessed as unclear risk of bias, and as high risk when one or more items being assessed as high risk of bias [26].

### Summary measures and approach to synthesis

The original outcome data regarding the efficacy of vibration were extracted and had been planned to undergo statistical pooling when the heterogeneity of primary studies was acceptable. When conducting meta-analysis failed, a qualitative summarization of evidences was adopted. The quality of synthesized evidence was assessed using Grading of Recommendations Assessment, Development and Evaluation (GRADE) system [27].

## Results

### Study selection and characteristics

**Fig. 1:**
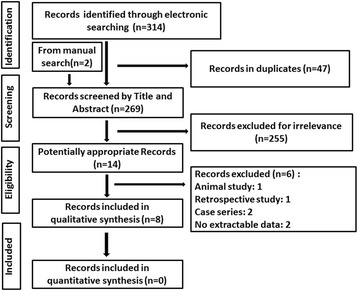
PRISMA flow diagram of study selection

The details of search results are depicted in a PRISMA flow-diagram (Fig. 1). Three hundred fourteen studies were identified in the electronic databases, and two additional records were found through manual search. After removal of the duplicates, a total of 269 citations were screened based on title and abstract. Subsequently, full texts of the 14 reserved studies were retrieved for assessment referring to the inclusion and exclusion criteria. Finally, eight studies were included in the review [16–23]. The Cohen’s-Kappa coefficient was used to measure inter-examiner agreement in the study selection process [24]. The kappa score was 0.86, indicating the interrater bias was low [28].

The general information of included studies was presented in Table 2. Out of the eight studies, seven were RCTs [16–19, 21–23] and one was demarcated as CCT [20]. Three publications were different parts of a same clinical trial, and were all included in this review since each of them reported an important outcome of vibration-assisted orthodontics respectively (tooth movement velocity, pain and discomfort, and root resorption) [17–19]. The sample size of single primary study ranged from 15 to 81. A total of 305 participants were involved in present review, among which 149 subjects underwent vibrational stimulus and 171 patients were assigned into sham/control group (15 patients were involved in split-mouth design). The priori sample size calculation was conducted in seven studies [16–19, 21–23].

**Table 2:** Overview of included studies

Study ID	Study design	Origin	Participants description	grouping	Vibration device	Type of tooth movement
Miles (2012) [16]	RCT	Australia	*n* = 66; F:40; M:26; Age:11–15	E:M:12;F:21;Age:13.0 ± 0.18y; C:M:14; F:19;Age:13.1 ± 0.18y	Tooth Masseuse (111 Hz, 0.06 N)	Alignment of the 6 mandibular anterior teeth (3–3)
Pavlin (2015) [23]	RCT	USA	*n* = 45; Age:12–40	E:*n* = 23; C:*n* = 22	AcceleDent (30 Hz, 0.25 N)	Retraction of maxillary canine or maxillary anterior teeth (3–3).
Woodhouse (2015a) [18]^a^	RCT	UK and Germany	*n* = 81; M:40, F:41. Age: 14.06 ± 1.7y	E:*n* = 29; Age: 13.9 ± 1.6y Sham: *n* = 25; Age: 14.1 ± 1.9y C: n = 27; Age: 14.4 ± 1.8y	AcceleDent (30 Hz, 0.25 N)	Alignment of the mandibular arch in comprehensive treatment
Woodhouse (2015b) [17]^a^	RCT	UK and Germany	n = 81; M:40, F:41. Age: 14.1 ± 1.7y	E:n = 29;M:15, F:14; Age: 13.9 ± 1.6y Sham: n = 25; M: 13, F:12 Age:13.8 ± 1.7y C: n = 27; M:12, F:15; Age: 14.4 ± 1.9y	AcceleDent (30 Hz, 0.25 N)	Alignment of the mandibular arch in comprehensive treatment
Leethanakul (2016) [20]	CCT	Thailand	*n* = 15; M:4; F:11 Age:19-25y	Split-mouth design, the experimental tooth (right or left) was determined randomly.	A electronic toothbrush (Colgate) with a vibrating head (125 Hz)	Retraction of maxillary canines after complete alignment.
Lobre (2016) [21]	RCT	USA	*N* = 58	E:n = 29 C:*n* = 29	AcceleDent(30 Hz, 0.25 N)	Alignment in comprehensive treatment
Miles (2016) [22]	RCT	Australia	*n* = 40; M:14; F:26 Age:12.8 ± 1.3y	E:M:6; F:14; Age: 12.7 ± 1.2y C:M:8; F:12; Age: 13.0 ± 1.5y	AcceleDent(30 Hz, 0.25 N)	Alignment of the 6 mandibular anterior teeth (3–3)
DiBiase (2016) [19]^a^	RCT	UK and Germany	n = 81;	E:n = 29 Sham: n = 25 C: *n* = 27	AcceleDent (30 Hz, 0.25 N)	Alignment in comprehensi9ve treatment

^a^The three publications are different parts of a same clinical trial

*E* experimental (vibration) group, *C* control group

### Risk of bias within studies

**Fig. 2:**
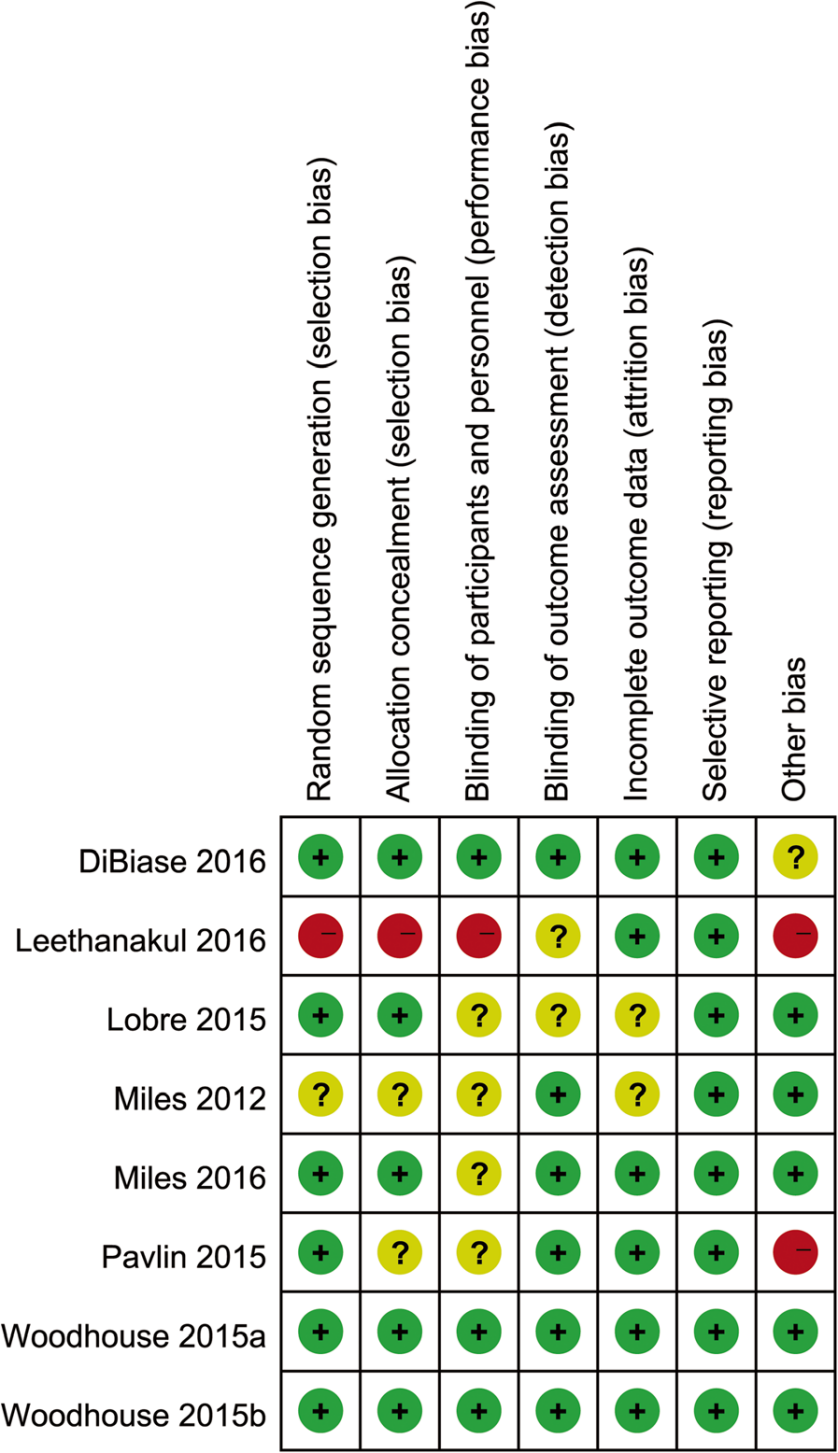
Risk of bias summary for included studies

The results of risk of bias assessment are summarized in Fig. 2. Among the eight studies, two studies (from a same clinical trial) were assessed as low risk of bias [17, 18], four were rated as unclear risk of bias [16, 19, 21, 22], and two were high risk of bias [20, 23]. Sixed studies adequately addressed random sequence generation [17–19, 21–23], and five of them were considered as reliable in allocation concealment [17–19, 21, 22]. The items regarding randomization and allocation concealment in CCT were set by default as high risk [20]. Since the use of vibrational devices, participants could not be blinded, which might influence the visual analogue scale scoring for pain perceptions. Therefore four studies were assessed as unclear risk for performance bias [16, 21–23]. Woodhouse et al. included a group of subjects using sham appliance and adequately considered the blind for participants regarding the allocation of functional and sham group [17]. Thus it was assessed as being free of performance bias [17]. Two studies did not report the details of missing data of drop-outs, thus were considered as unclear risk for attrition bias [16, 21]. High risk of other bias was present in two studies since split-mouth design and sponsorship from manufacturer of vibrational devices respectively [20, 23]. Unclear risk of other bias was detected in one study since root resorptions were evaluated using periapical radiographs [19].

### Description of interventions

All the included studies investigated the effects of vibrational stimulus on orthodontic treatment, but the procedures of vibrational stimulus and tooth movement differed (Tables 2, 3). One study adopted Tooth Masseuse that provided a vibrational force of 111 Hz and 0.06 N for 20 min per day [16]. Six studies used AcceleDent (OrthoAccel Technologies, Inc., Bellaire, TX) which delivers a vibrational force of 0.25 N with a frequency of 30 Hz [17–19, 21–23]. The other one study employed an electrical toothbrush with a vibration head (125 Hz) [20]. When concerning the type of tooth movement, six studies investigated mandibular teeth alignment [16–19, 21, 22] and the other two focused on maxillary canine retraction (Table 2) [20, 23].

**Table 3:** Characteristic of interventions and results

Study ID	Vibration details	Orthodontic treatment	Follow-ups	Tooth movement measurement	Primary result	Secondary result
Miles (2012) [16]	A vibrational device (Tooth Masseuse) provides vibration (111 Hz, 0.06 N) for 20 min per day.	Level and align the lower anterior teeth using 0.018*0.025-in. bracket slot system and 0.014 in. NITI wire in cases without extraction in lower arch or impacted teeth.	10 weeks (assessed at 5, 8 and 10 weeks after commencement)	Reduction in the irregularity assessed by Little’s Irregularity Index.	No differences in irregularity were observed at 5, 8 and 10 weeks after commencement between 2 groups.	No differences pain levels were observed at any time points between 2 groups.
Pavlin (2015) [23]	A vibrational device (AcceleDent) provides vibration (30 Hz, 0.25 N) for 20 min per day.	After complete alignment, maxillary canines or all 6 anterior teeth were retracted using miniscrews under force of 180 g, in patients that had maxillary first premolars extracted	Entire space closure	Measured using a digital caliper in patients’ mouth.	The rate of canine retraction was higher in the AcceleDent group (1.16 mm/month) than the control group (0.79 mm/month).	No differences in root resorption and pain levels were observed between 2 groups.
Woodhouse (2015a) [18]^a^	A vibrational device (AcceleDent) provides vibration (30 Hz, 0.2 N) for 20 min per day.	Level and align the mandibular arch using sequential arch wires (from 0.014-in NITI wire to 0.019*0.025-in stainless wire) in patients that had mandibular premolars extracted.	Entire alignment (from 0.014-in NITI wire to 0.019*0.025-in stainless wire)	Difference in the irregularity (Little’s irregularity index) divided by the number of days between measurements.	No significant difference in the rate of tooth movement or amount of required time among 3 groups.	–
Woodhouse (2015b) [17]^a^	A vibrational device (AcceleDent) provides vibration (30 Hz, 0.2 N) for 20 min per day.	Level and align the mandibular arch using 0.014-in NiTi and 0.018-in NiTi wire in patients that had mandibular premolars extracted.	Initial alignment from 0.014-in NiTi wire to 0.018-in NiTi wire.	Difference of irregularity index of casts taken at placement of 0.014-in and 0.018-in NiTi wire divided by number of days between 2 measurements	No significant difference in the rate of tooth movement in the initial alignment phase was observed among 3 groups.	The maximum and mean pain intensity in the first week after placement of 0.014-in and 0.018-in NiTi wire similar among 3 groups
Leethanakul (2016) [20]	A electronic toothbrush (Colgate) with a rotating and vibrating head (125 Hz), which is used to provide mechanical vibration for a minimum of 5 min 3 times per day.	After complete alignment, the maxillary canine was retracted using elastomeric chain on the buccal and palatal side, under force of approximate 60 g, in patients that had maxillary first premolars extracted	2 months	Difference in the distance between canine and third rugae among consecutive models, assessed using digital caliper.	The accumulative amount of tooth movement was greater for the experimental canine (2.85 ± 0.17 mm) than for the control group (1.77 ± 0.11 mm).	–
Lobre (2016) [21]	A vibrational device (AcceleDent) provides vibration (30 Hz, 0.25 N) for 20 min per day.	Level and align dentitions, and returned per months.	4 months	–	–	Vibrational stimulus significantly reduced the intensity of overall pain and biting pain.
Miles (2016) [22]	A vibrational device (AcceleDent) provides vibration (30 Hz, 0.25 N) for 20 min per day.	Participants were Class II patients with maxillary premolars extracted and receiving comprehensive treatment in both archs. The dentitions were aligned using a 0.014-in thermal NiTi wire in the whole experiment.	10 weeks	Arch perimeter and irregularity of mandibular anterior teeth (3–3) assessed using Alginate impressions	No significant differences in anterior arch perimeters and irregularity were observed at 5, 8 or 10 weeks after commencement between 2 groups.	No significant difference of pain intensity in the first week after appliance placement.
DiBiase (2016) [19]^a^	A vibrational device (AcceleDent) provides vibration (30 Hz, 0.2 N) for 20 min per day.	Level and align using sequential arch wires (from 0.014-in NITI wire to 0.019*0.025-in stainless wire) in patients that had mandibular premolars extracted.	Entire alignment (from 0.014-in NITI wire to 0.019*0.025-in stainless wire)	–	–	No significant difference in the external root resorptions of maxillary right central incisors among 3 groups.

^a^The three publications are different parts of a same clinical tria

### Results of individual studies and data synthesis

Since the substantial differences in vibration parameters, type of tooth movement and outcome measurements among included studies (Tables 2, 3), it is difficult to quantitatively combine the original outcome. Thus, the results of included trials were summarized qualitatively. The quality of synthesized evidence for each outcome was assessed using GRADE approach and presented in Table 4.

**Table 4:** GRADE assessment for quality of evidence

Patient or population: patients with orthodontic treatment Settings: RCT and CCT Intervention: vibrational stimulus						
Outcomes	Illustrative comparative risks (95% CI)		Relative effect(95% CI)	No of Participants(studies)	Quality of the evidence(GRADE)	Comments
	Assumed risk	Corresponding risk				
	Control	Vibrational stimulus				
rate of OTM in alignment	See comment	See comment	Not estimable	187(3 studies)	⊕⊝⊝⊝very low^a,b,c^	vibrational stimulus did not increase tooth movement in alignment
rate of OTM in canine retraction	See comment	See comment	Not estimable	60(2 studies)	⊕⊝⊝⊝very low^b,d,e^	vibrational stimulus increased the rate of canine retraction

^a^Unclear risk in random sequence generation, allocation concealment, blinding of participants, incomplete reporting and other bias

^b^The procedure of orthodontic treatment and vibrational stimulus and follow-up durations varied

^c^Only three studies with limited sample size (*n* = 187) were included

^d^High risk of bias in randomization, blinding and other bias

^e^Only two studies with limited sample size (*n* = 60) were included

#### Rate of tooth movement in alignment

Miles et al. compared the reduction of irregularity index of mandibular anterior teeth between patients receiving vibrational stimulus (Tooth Masseuse) or not. No significant difference of mean irregularity index reduction between two groups was detected at 5 (50% vs 45%), 8 (61% vs 61%) or 10 weeks (65% vs 69%) after commencement [16]. Thereafter, Miles et al. investigated the effectiveness of AcceleDent device in another trial, and observed similar rates of anterior lower teeth alignment between experimental and control groups [22]. Woodhouse and DiBiase et al. conducted a RCT involving patients treated with AcceleDent vibrational device, nonfunctional device or orthodontic treatment only, and reported results of alignment rate in two publications. One study showed that the time required for initial and final alignment of mandibular dentition were similar among three groups (initial alignment: 56.3 vs 59.8 vs 61.0 day; final alignment: 210.2 vs 217.5 vs 200.7 day) [18]. Similarly, the other study demonstrated no difference in the average rate of initial alignment was observed (0.10 ± 0.05 mm vs 0.11 ± 0.06 mm vs 0.10 ± 0.05 mm) [17]. These two publications were considered as one study for constructing body evidence (Table 4). According to GRADE, the quality of evidence on the effect of vibrations on alignment is assessed as very low (Table 4).

#### Rate of tooth movement in canine distalization

Pavlin et al. [23] reported the average rate of maxillary canine retraction was significantly enhanced by using AcceleDent device for 20 min per day (1.16, 95% CI: 0.86–1.45 mm/month vs 0.79, 95%CI: 0.49–1.09 mm/month). Leethanakul et al. [20] showed the accumulative distance of canine distalization on the quadrant receiving stimulation from a vibrating head (125 Hz) of electronic toothbrush was enhanced compared to the control side (2.85 ± 0.17 mm vs 1.77 ± 0.11 mm). The quality of evidence supporting the efficacy of vibrations on canine retraction is assessed as very low (Table 4).

#### Pain and discomfort

Five studies evaluated the effects of vibrational stimulus on pain intensities using visual analogue scale (VAS). Miles et al. found the use of vibratory device (Tooth Masseuse) did not reduce pain perceptions at 6-8 h (39.6 ± 25.8 mm vs 40.4 ± 20.8), 1 day (47.6 ± 24.5 mm vs 41.5 ± 27.2), 3 days (19.9 ± 15.5 mm vs 18.8 ± 18.5) and 7 days (5.5 ± 7.8 mm vs 4.0 ± 6.3) after placing appliances [16]. Similar results were observed in another RCT conducted by Miles et al., which adopted AcceleDent to produce vibrational stimulus [22]. Woodhouse et al. found either the maximum or mean pain intensities in the first week after placement of 0.014-in. and 0.018-in. NiTi archwires were similar among the vibration (AcceleDent), sham (nonfunctional AcceleDent) and control group [17]. Pavlin et al. also reported that no significant difference in pain or discomfort was detected in participants treated with vibrations or not. On contrary to the foregoing studies which suggested that vibrational stimulus did not influence pain levels, Lobre et al. evaluated the biting and overall pain in the first 4 month of treatment, and found that patients treated with vibration (AcceleDent) perceived both biting (*P* = 0.003) and overall pains (*P* = 0.002) of lower intensities during the whole study period [21].

#### Root resorption

DiBiase et al. evaluated the right maxillary central incisor lengths before and after alignment using periapical radiographs, and found the orthodontically induced inflammatory root resorption was not affected by application of supplemental vibrational force [17]. Similarly, Pavlin et al. reported no difference in root resorption after canine retraction between patients using vibrational device (AcceleDent) or not [23].

## Discussion

To our knowledge, this is the first systematic review addressing the efficacy of vibrational stimulus to accelerate OTM. This review included eight prospective clinical trials comprising an overall sample of 305 patients. The heterogeneity in methodology and non-comparability of outcome measures in retrieved publications prevented a quantitative synthesis from being performed. Therefore, we collected, appraised and qualitatively synthesized the currently available literatures to provide evidence regarding this issue.

Four studies [16–18, 22] in this review investigated the rate of tooth movement in alignment, two of which were the different parts of a same RCT [17, 18]. All of the four publications reported that the use of vibratory device could not enhance the velocity of tooth movement during alignment (Table 3). However, this result should be interpreted with caution since following reasons. Firstly, only four publications (three clinical trials) are included for this outcome, and the methodological flaws are noteworthy (Fig. 2). Secondly, vibrational stimuli are different in the four studies (Tables 2, 3). Thirdly, Studies reported by Miles et al. [16, 22] focused on the alignment of lower anterior teeth in patients without extraction in mandible while the other two studies reported by Woodhouse et al. [17, 18] aligned the whole mandibular dentition in patients that had mandibular first premolars extracted (Table 3). Due to the reasons above, quality of this evidence was assessed as very low quality referring to GRADE guidance (Table 4) [27].

Two studies [20, 23] evaluated the effects of vibration on canine distalization in patients that had maxillary first premolar extracted (Tables 2, 3). Results of the two studies advocated the advantage of vibrational forces in acclerating canine retraction (Table 3). Leethanakul et al. detected enhanced IL-1β secretion in gingival crevicular fluid in quadrant receiving vibrational stimulus compared to the control quadrant [20]. IL-1 could induce RANKL expression in osteoblasts and periodontal ligament cells, and also promote the differentiation of pre-osteoclast [29]. Interestingly, a well-designed animal study indicated that vibration could promote osteoclast formation via enhancing RANKL expression in periodontal tissue and thus facilitate alveolar bone remodeling and lead to faster tooth movement [13]. These studies suggested that vibrational stimulus could accelerate OTM through promoting osteoclast formation and alveolar bone remodeling. However, current evidence supporting the effectiveness of vibration on accelerating canine distalization is of very low quality, mainly due to the lack of high-quality primary studies and methodological heterogeneity (Table 4).

Vibratory stimulations have been proved to reduce pain perceptions in different fields [30, 31]. Five studies in this review investigated effects of vibrations on orthodontic pain (Table 3). However, the reliability of these results is questionable since the absence of blinding to participants could influence the VAS scoring for pain levels. Only one study was free of this bias since it included participants intervened with identical non-functional device and concealed the allocation of functional and non-functional group, which suggested that vibration did not influence pain levels [17]. Anyway, no conclusion concerning the effects of vibration on pain and discomfort could be drawn based on current information.

Root resorption is one of the main complications in orthodontic treatment [32]. DeBiase et al. assessed the changes of root lengths after orthodontic treatment using periapical radiographs [19]. However, film radiographs could only provide information of apical root resorptions. The resorptions occurring at other sites on root could be underestimated [33]. Another study observed no impact of vibrations on root resorptions. Nevertheless, no details of measurements and outcomes were reported [23]. In general, no reliable result on root resorption is available yet.

OTM is the consequence of tissue remodeling within periodontium induced by external forces [34]. Most of the current adjunctive interventions, like corticotomy, enhance the rate of tooth movement by promoting alveolar bone remodeling [35]. The anabolic effects of supplemental vibrational therapy on bone metabolism have been long recognized [36]. Its effectiveness in promoting suture growth and remodeling in craniofacial region has also been identified [37]. A recent study indicates that vibration could accelerate OTM through promoting alveolar bone remodeling [13]. However, another experiment found that mechanical vibration did not increase the number of osteoclasts or rate of tooth movement [38]. It should be noted that distinguished difference of vibration frequency exists in these two animal studies (60 vs 5–20 Hz), indicating that vibratory stimulus could act in a frequency-dependent manner. Therefore, future clinical trials should be carried out to explore the optimal protocols of vibratory forces for accelerating OTM.

Although this systematic review was performed carefully following normalized procedures, several limitations which deserved further discussion still existed. First, the shortage of high-quality clinical trials is evident. Though a comprehensive literature search was performed, only eight studies were included in this review. Future well-designed studies are needed to obtain a more reliable conclusion. Second, the methodological heterogeneity and non-comparability of original outcomes could bias the qualitative summarization of this review. Third, the publication bias has not been investigated since the weak statistical power when included publications are limited [39]. Fourth, the language restriction in literature search could have introduced bias into this review.

## Conclusions

Based on current information, weak evidence suggests that vibrational stimulus is effective for accelerating tooth movement in canine retraction but not in the alignment phase. The effects of vibration on pain intensity and root resorption during orthodontic treatment are inconclusive. There is a need for well-designed randomized controlled trials to obtain more reliable results.

## Abbreviations

CCT: Controlled clinical trials
CENTRAL: Cochrane Central Register of Controlled Trials
GRADE: Grading of Recommendations Assessment, Development and Evaluation
MeSH: Medical Subject Headings
OTM: Orthodontic tooth movement
PRISMA: Preferred Reporting Items for Systematic Reviews and Meta-analyses
RCT: Randomized controlled trials
SIGLE: System for Information on Grey Literature in Europe
VAS: Visual analogue scale
